# When Infection Masquerades as Malignancy: Fatal Basidiobolomycosis Presenting as Suspected Rhabdomyosarcoma in a Child

**DOI:** 10.1155/crpe/8135826

**Published:** 2026-06-16

**Authors:** Emmanuella Amoako, Bashiru Babatunde Jimah, Lily Gloria Tagoe, Rebekah Ruth Taylor, Lydia Awuku, Percival Lomotey, Yemah Bockarie, Kofi Ulzen-Appiah, Benjamin Dabo Sarkodie, Loretta Inkoom

**Affiliations:** ^1^ Department of Paediatrics and Child Health, Cape Coast Teaching Hospital, Cape Coast, Ghana, ccthghana.org; ^2^ Department of Radiology, School of Medical Sciences, University of Cape Coast, Cape Coast, Ghana, ucc.edu.gh; ^3^ Department of Paediatrics and Child Health, Korle Bu Teaching Hospital, Accra, Ghana, kbth.gov.gh; ^4^ Department of Pathology, Cape Coast Teaching Hospital, Cape Coast, Ghana, ccthghana.org; ^5^ Department of Radiology, School of Medical Sciences, University of Ghana, Accra, Ghana, ug.edu.gh; ^6^ Department of Radiology, 37 Military Hospital, Accra, Ghana

## Abstract

**Background:**

Basidiobolomycosis is an uncommon fungal infection caused by *Basidiobolus ranarum*, primarily impacting immunocompetent children in tropical areas. Its indolent presentation and radiologic mimicking to soft tissue cancers often result in misdiagnosis and delayed antifungal treatment.

**Case Presentation:**

We report the case of a 10‐year‐old child from a remote farming community in Ghana who had a 2‐year history of a progressively increasing abdominal wall mass, severe malnutrition, and respiratory distress. The initial computed tomography scan suggested rhabdomyosarcoma. Laboratory tests showed severe anemia, hypoalbuminemia, and marked eosinophilia. Histopathology revealed pronounced granulomatous inflammation characterized by multinucleated giant cells and broad septate fungal hyphae, as evidenced by PAS and Grocott stains, thereby confirming basidiobolomycosis. Sadly, the child’s condition deteriorated rapidly, with resultant demise prior to the commencement of antifungal treatment.

**Conclusion:**

Basidiobolomycosis could mimic a pediatric soft tissue sarcoma on imaging, particularly in endemic regions. Early clinical suspicion, early detection of eosinophilia, and timely biopsy are crucial to prevent diagnostic delays and enhance outcomes, particularly in resource‐limited healthcare systems.

**Learning Points**
• Basidiobolomycosis may mimic soft tissue sarcoma on imaging.
• Peripheral eosinophilia is an important diagnostic clue.
• Early biopsy is critical in chronic pseudotumoral masses.
• Delayed diagnosis in resource‐limited settings increases mortality risk.

## 1. Introduction

Basidiobolomycosis is a rare but potentially fatal fungal infection caused by *Basidiobolus ranarum*, an environmental saprophyte in the order of Entomophthorales found in decaying vegetation and soil [1–3]. The infection predominantly affects immunocompetent children and adolescents [4], typically involving the trunk and extremities following minor traumatic inoculation [2, 5]. Although cutaneous disease is most recognized, systemic involvement of the lung, gastrointestinal tract, and liver has become increasingly reported [1, 6].

Epidemiologically, basidiobolomycosis occurs mainly in tropical and subtropical environments and has been documented in regions across the Middle East, Africa, and South America [1, 2]. Environmental exposure, particularly farming, or contact with decaying organic matter appears to play an important role in disease acquisition, likely reflecting the saprophytic nature of the fungus and its ecological niche [1, 2, 4].

Clinically, the disease presents as a slowly expanding, firm “wood‐like” plaque or mass, often accompanied by peripheral eosinophilia. Because radiologic and clinical features may simulate soft tissue sarcomas such as rhabdomyosarcoma, misdiagnosis and delays in antifungal therapy are common [1, 7]. Early biopsy and histopathologic evaluation are therefore essential to avoid inappropriate oncologic workup or treatment.

We report a fatal case of basidiobolomycosis in a 10‐year‐old boy from a rural farming community in Ghana, initially suspected to have rhabdomyosarcoma based on imaging characteristics.

## 2. Case Report

A 10‐year‐old male from a farming community presented with a 2‐year history of a progressively enlarging abdominal mass and severe malnutrition. Initial CT imaging demonstrated a heterogeneous lesion involving the left lateral abdominal wall measuring 14 × 10 × 6 cm with partial intra‐abdominal extension. Areas of low attenuation suggested necrosis (Figure 1). The radiologic differential diagnosis included rhabdomyosarcoma. Laboratory evaluation revealed microcytic hypochromic anemia, hypoalbuminemia, and peripheral eosinophilia.

**FIGURE 1:**
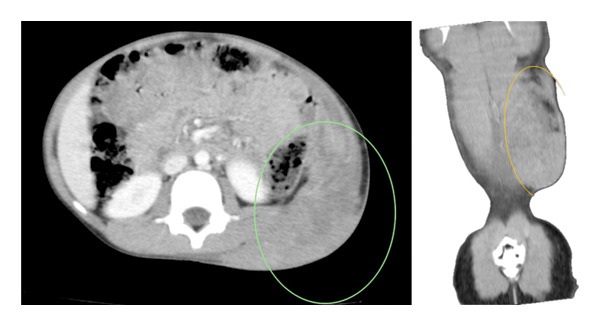
Contrast‐enhanced axial and coronal CT scan images showing homogeneously enhancing soft tissue mass in the left lateral abdominal wall (green circle on the axial and yellow circle on the coronal) with nonenhancing areas of necrosis or hemorrhage. Subtle intra‐abdominal extension noted.

Further history revealed that he had presented 2 years prior and had the initial CT imaging done but was lost to follow‐up. He then returned with a worsening abdominal mass, progressive weight loss, and breathlessness. Physical examination revealed a poorly nourished child with dullness to percussion and marked decreased air entry over the left lung. Abdominal examination showed a hyperpigmented, indurated, “wood‐like” mass extending from the left flank to the hypochondrium. Chest radiograph confirmed a massive left pleural effusion (Figure 2). Chest tube drainage was performed, yielding approximately 500 mL of serous fluid. Cytologic examination of the pleural fluid demonstrated a mixed inflammatory infiltrate consisting predominantly of neutrophils, with lymphocytes, plasma cells, and occasional eosinophils. Repeat cross‐sectional imaging was not performed due to financial constraints. Serologic screening for HIV and hepatitis A and B viruses was negative.

**FIGURE 2:**
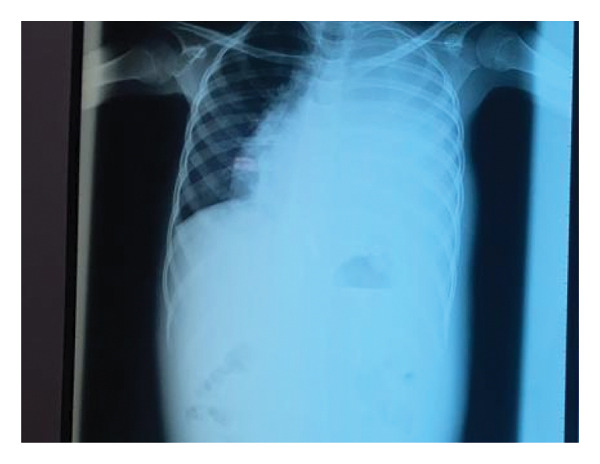
Posteroanterior radiograph of the chest shows massive left pleural effusion with tracheal and mediastinal deviation to the right. The right lung is clear. Subtle soft tissue swelling along the left lateral abdominal wall.

A biopsy of the abdominal wall lesion was taken, and histopathological analysis showed florid granulomatous inflammation with multinucleated giant cells and numerous thin‐walled, broad septate hyphae, some engulfed by giant cells (Figure 3). Special stains (PAS and Grocott) highlighted the hyphae, confirming basidiobolomycosis (Figure 4). Fungal cultures were not done due to resource limitations.

**FIGURE 3:**
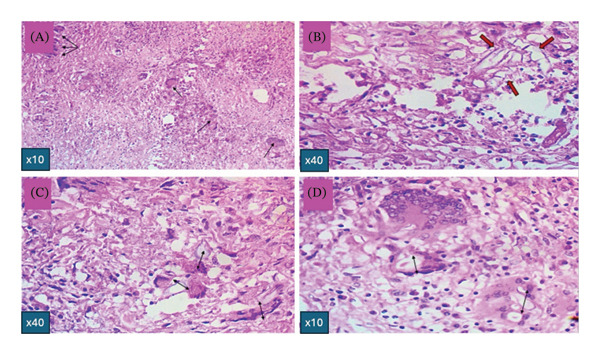
(A) Florid granulomatous inflammation with multinucleate giant cells (black arrows) within sclerotic stroma (X 10). (B) Thin‐walled broad septate hyphae (red arrows) (X 40). (C, D) Multinucleate giant cells engulfing hyphae (double black arrows) (X 40).

**FIGURE 4:**
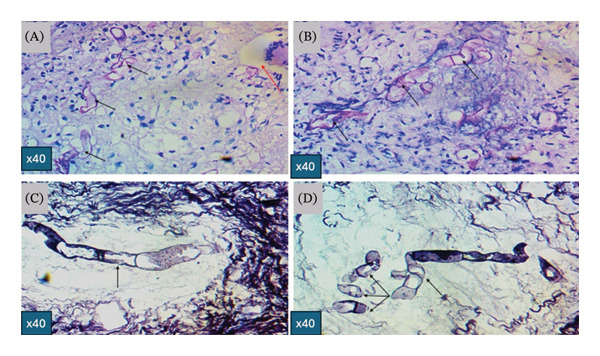
(A, B) PAS stain highlighting thin‐walled broad septate hyphae (red arrows). (C, D) Grocott stain highlighting thin‐walled broad septate hyphae (black arrows) (X 40).

Unfortunately, the child’s condition deteriorated and he died shortly thereafter before antifungal medications could be started. Postmortem examination was declined by his caregivers.

## 3. Discussion

Basidiobolomycosis remains an underrecognized fungal infection in many low‐ and middle‐income settings, in part due to its rarity, nonspecific presentation, and radiologic mimicry of malignancy. The indolent course and firm “wooden” texture of the lesion often create a strong clinical and radiologic impression of a soft tissue sarcoma, particularly rhabdomyosarcoma. This overlap in presentation has been reported in multiple case studies, underscoring the need for clinicians and pathologists to maintain awareness of fungal mimics of malignancy when evaluating pediatric masses, especially in children with agricultural or soil exposure.

In our patient, the initial CT scan suggested a malignant soft tissue tumor due to heterogeneous enhancement, areas of necrosis, and intra‐abdominal extension. Similar radiologic features have been described in the literature and contribute to diagnostic delays, which can be consequential since early initiation of antifungal therapy has been shown to improve outcomes. Peripheral eosinophilia, present in this child, is a valuable laboratory clue and has been a consistent feature across reported cases [2, 6]. Although nonspecific, eosinophilia in the context of a chronic pseudotumoral lesion should expand the differential to include basidiobolomycosis, mucormycosis, and helminthic infections.

Histopathology remains the cornerstone of diagnosis. Granulomatous inflammation with multinucleated giant cells and broad fungal hyphae are characteristic findings. Special stains such as PAS and Grocott enhance diagnostic certainty. While culture confirmation allows species‐level identification, access and yield may be limited in certain settings [2, 5]. In our case, diagnosis relied on morphology and special stains alone, a scenario that reflects the realities of many resource‐limited health systems. In the absence of culture, histomorphological features are critical in differentiating basidiobolomycosis from other fungal infections such as mucormycosis. *Basidiobolus* typically demonstrates broad, sparsely septate hyphae associated with a prominent granulomatous and eosinophil‐rich inflammatory response, often with the Splendore–Hoeppli phenomenon. In contrast, Mucorales species characteristically exhibit broad, ribbon‐like aseptate hyphae with angioinvasion and tissue necrosis, usually in immunocompromised hosts. The absence of angioinvasion and the presence of granulomatous inflammation in our case favored basidiobolomycosis.

The disease is generally responsive to prolonged antifungal therapy, including itraconazole and potassium iodide, and surgical resection may be useful in localized disease [1, 2, 4, 8]. Reported survival is favorable when treatment is instituted early. However, mortality increases with delayed diagnosis, malnutrition, visceral involvement, and barriers to care, all of which contributed to the adverse outcome in this case [1, 2, 5]. The patient’s prolonged course, financial limitations, and loss to follow‐up highlight how social determinants influence the trajectory of uncommon infections [2, 8]. These factors are often underrepresented in the literature but are highly relevant in low‐ and middle‐income countries, where delayed presentations are common.

Given the potential for misdiagnosis, basidiobolomycosis should remain in the differential diagnosis of chronic, firm abdominal or truncal masses with associated eosinophilia in children from farming regions or those with environmental exposure. Prompt biopsy is essential, both to exclude malignancy and to enable timely targeted therapy that improves clinical outcomes.

## 4. Conclusion

Basidiobolomycosis, although curable, may be fatal if misdiagnosed. Its ability to mimic soft tissue sarcomas on imaging underscores the need for early clinical suspicion, particularly in children from agricultural communities presenting with indolent “wood‐like” masses and peripheral eosinophilia. Early biopsy and histopathologic confirmation are essential to prevent delays in initiating appropriate antifungal therapy. In low‐resource settings, limited access to repeat imaging, fungal cultures, antifungal medications, and subspecialty pathology services may further compound diagnostic delays. This case illustrates how structural inequities intersect with rare infectious diseases to worsen outcomes.

## Funding

No funding was received for this manuscript.

## Ethics Statement

Ethical approval was waived by the Cape Coast Teaching Hospital Institutional Review Board, as this study is a single case report. The study was conducted in accordance with institutional guidelines and the principles of the Declaration of Helsinki.

Written informed consent for publication of this case report and accompanying images was obtained from the patient’s parent/legal guardian.

## Conflicts of Interest

The authors declare no conflicts of interest.

## Data Availability

The data that support the findings of this study are available on request from the corresponding author. The data are not publicly available due to privacy or ethical restrictions.
